# Bridging the Digital Divide: Applying Digital Tools to Improve Cardiovascular Health in Underrepresented Communities

**DOI:** 10.1016/j.jacadv.2023.100587

**Published:** 2023-09-01

**Authors:** Trent Johnson, Michael Chilazi, Nino Isakadze, Karan Desai, Erin M. Spaulding, Amit Goyal, Dan Ambinder, Garima Sharma, Dipti Itchhaporia, Seth S. Martin, Francoise A. Marvel

**Affiliations:** a Division of Cardiology, Department of Medicine, Digital Health Innovation Laboratory, Ciccarone Center for the Prevention of Cardiovascular Disease, Johns Hopkins University School of Medicine, Baltimore, Maryland, USA; b Division of Cardiology, Massachusetts General Hospital, Boston, Massachusetts, USA; c Division of Cardiology, Department of Medicine, Johns Hopkins University, Baltimore, Maryland, USA; d Johns Hopkins University School of Nursing, Baltimore, Maryland, USA; e Department of Cardiovascular Medicine, Cleveland Clinic, Cleveland, Ohio, USA; f Division of Cardiology, University of Maryland, Baltimore, Maryland, USA; g Inova Heart and Vascular Institute, Falls Church, Virginia, USA; h Division of Cardiology, University of California, Irvine, California, USA

**Keywords:** cardiovascular disease, digital health, disparities, primary prevention, underserved, young adults

## Background

Cardiovascular disease (CVD) disparities present a major challenge for the global cardiology community. Health disparities continue to account for a 30% higher mortality rate from CVD among Black communities compared to non-Hispanic White Americans.^1^ For example, in Northeast Baltimore, where many members of this author group live and practice, zip codes separated by single-digit miles experience double-digit differences in life expectancy.^2^ Importantly, forces shaping these health care disparities begin early in life leading to disproportionately higher prevalence of obesity, hypertension, diabetes, and dyslipidemia among young minorities.^3^ The transition from adolescence to adulthood represents a critical juncture in promoting long-term cardiovascular health among underrepresented communities.

Digital health technology offers a powerful tool to connect with young audiences to promote awareness of, and engagement in, cardiovascular health. Rising popularity in smartphones and wearables among young adults has fueled substantial investment in this sector.^4^ Additionally, social media platforms are becoming increasingly popular forums where patients and health professionals are accessing cardiovascular health information.^5^ The COVID-19 pandemic only accelerated the evidence base for the impact of digital tools on patients.^6^ Digital tools thus serve as a promising vehicle to reach at-risk young adults to promote CVD prevention and improve health equity.^6^

**Figure 1:**
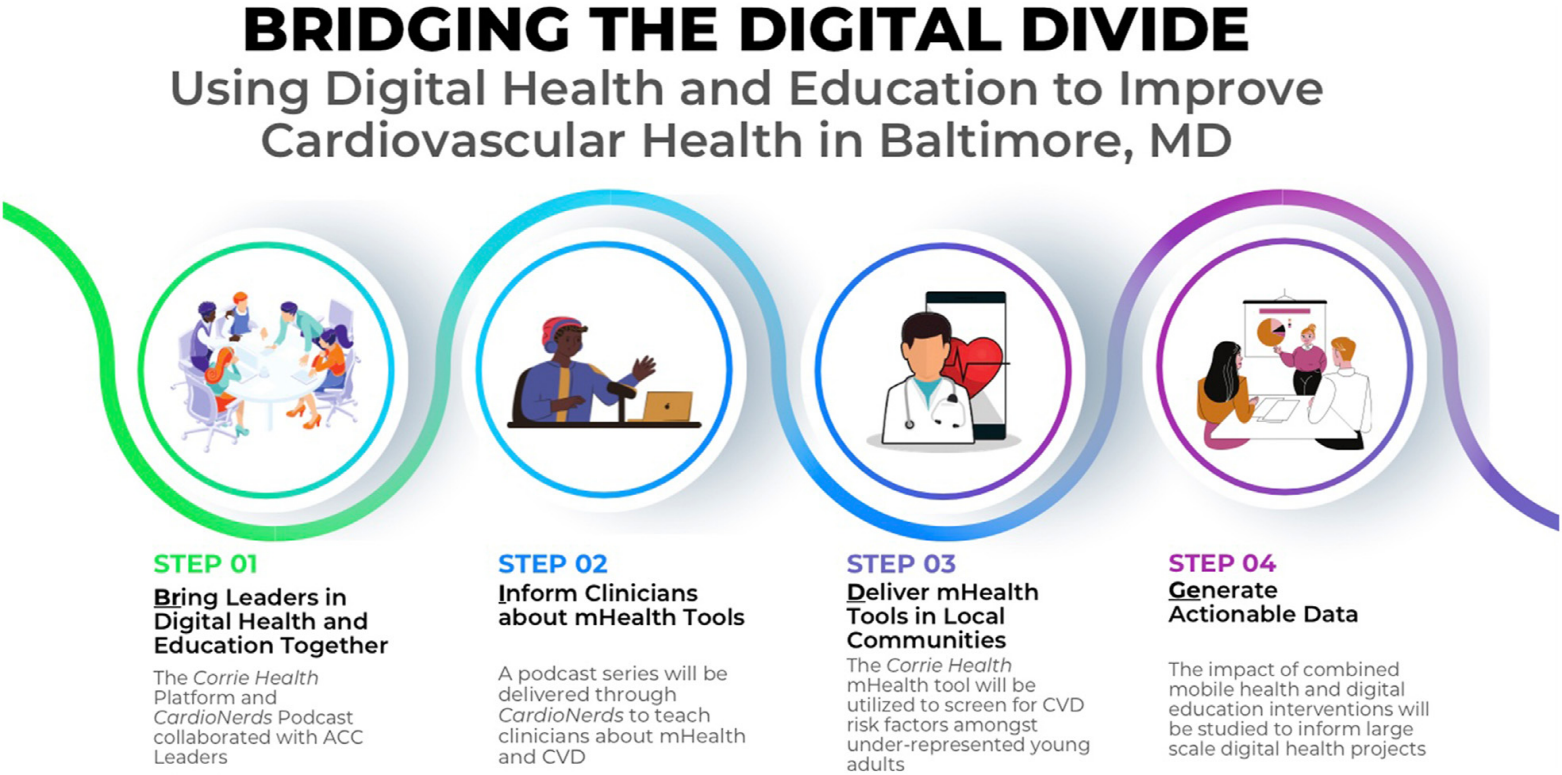
Key Steps to Bridging the Digital Divide Bridging the digital divide using digital health and education to improve cardiovascular health in Baltimore, Maryland: A 4-step intervention process involving collaboration between leaders in digital health and education, delivery of mobile health tools in local communities, and generation of actionable data to inform large-scale digital health projects. ACC = American College of Cardiology; CVD = cardiovascular disease.

Technological advancements, however, have come with cautionary guidance from thought leaders to avoid the creation of a “digital divide” whereby certain communities are excluded from digital innovations due to limited access, affordability, or familiarity.^7^ Socially conscious approaches are required for developing and implementing these technologies to reach disadvantaged communities and promote health equity by making health-related guidance and counseling more accessible. With this as our central goal, we embarked on a project supported by a Chapter-Section Grant from the American College of Cardiology Chapter of Maryland called “Bridging the Digital Divide” where we partnered with local communities and clinicians to harness digital tools to promote CVD prevention for vulnerable groups. Here we describe our implementation strategy and lessons learned to guide future initiatives (Figure 1).

## Partnering with the community

We partnered with students at Morgan State University (MSU), a Historically Black College and University, to cocreate an educational curriculum for CVD prevention relevant to at-risk young adults. We applied a Human Centered Design framework^8^ with end users in mind to structure 4 key phases of the project: ideate, create, feedback, and deploy.

### Ideate

Through conversations with MSU students on our leadership team, we identified an interest in understanding the risks of popular tobacco alternatives (eg, vaping, hookah, e-cigarettes) on cardiovascular health. Tobacco use remains a significant reversible risk factor for atherosclerotic cardiovascular disease in young adults, particularly in underrepresented minority groups.^3^ These products frequently target young adults and amongst the group at MSU, there was a strong interest in understanding the risks of vaping and benefits of cessation.

### Create

Once we identified the needs of the group, we created relevant educational content that included the prevalence of tobacco use in young adults, the health consequences of tobacco, tobacco alternatives (eg, vaping), and tobacco cessation resources. We collaborated with a premedicine research intern enrolled at MSU to better understand effective avenues to reach students on MSU’s campus with social media being one of the most cited tools for consumption of content related to health and well-being. With this knowledge, in collaboration with a professional videographer, we created a TikTok-style clip (<2 minutes) with engaging illustrative graphics to reinforce the importance of tobacco cessation.

### Feedback

We tested the acceptability of our digital content with MSU students by organizing an on-campus Health Hub event. Students viewed the educational content that we created and provided real time feedback regarding the perceived efficacy, messaging, and distribution. Thirteen students attended the Health Hub and on assessment of the intervention, 71% of students perceived this type of educational content delivery to be “very effective” and 29% perceived it to be “fairly effective.” Student surveys identified TikTok (50%), Twitter (38%), and YouTube Shorts (13%) as the preferred social media platforms to consume and share content.

### Deploy

We disseminated tobacco cessation content through various platforms including the Corrie mobile application (a digital health platform that provides education on risk factors for CVD and lifestyle modification,^9^ social media (eg, Twitter, Instagram, TikTok), podcasts, and on-campus health hubs based on MSU student feedback. Additionally, MSU students expressed interest in creating and sharing their own educational content centered on self-identified priority areas (eg, healthy eating, exercise, and alcohol use) under the mentorship of our team’s health care professionals. By transferring skills and empowering end users to be agents of change in their communities, CVD prevention efforts can be further amplified and impactful. These preferences should be considered when deciding on the highest-impact communication avenue with young adults in this demographic.

## Using podcasts to bridge the divide with health care professionals

We believed that our application of digital tools to promote CVD prevention among young adults would be further amplified by creating instructional content for health care professionals guiding them in this space. We collaborated with CardioNerds,^10^ a popular multimodality digital education platform, to make use of their podcast platform to create a series targeted at health care professionals to understand and harness digital health innovations in the care of patients. In doing so, we aimed to amplify the digital bridge connecting clinicians and patients. The series includes episodes covering the following topics: “How Digital Health is Changing Cardiovascular Disease Management,” “Using Digital Health to Reduce or Amplify Health Disparities,” “Barriers to Integrating Digital Health into Practice,” and “Tips for the Emerging Digital Health Innovator,” With over 30,000 Twitter followers and an audience spanning physicians, scientists, allied health professionals, nurses, and more, the CardioNerds series will serve to provide roadmaps to amplify initiatives similar to our on-the-ground work at MSU.

## Conclusions

The success of our digital health model stems from codesign with end users (youth and medical professionals) to break down the digital divide to promote health equity. Promoting equitable health care using digital health technology takes an integrative approach and involves medical professionals as well as the community. Cardiovascular health disparities disproportionately impact minority populations such as Black and Hispanic Americans. When creating digital content, it is imperative to partner directly with the end user during all phases of design (ie, ideate, create, feedback, and deployment). When disseminating targeted interventions, selecting the correct platform is the key. From our experience, we learned that young adults are motivated to close the gap on health disparities affecting their communities. In the future, we hope this comprehensive model which: 1) partners with end users to design and share digital content; and 2) engages health care providers using popular podcasts like CardioNerds to further these efforts can be used to inform creation of digital tools to reduce the risk of CVD in marginalized adults. Future directions and research efforts will focus on quantifying the outreach accomplished with digital tools (eg, capturing users reached through views, likes, and shares) and assessing to what degree content influenced knowledge and behavior. Ultimately, the model we created is scalable with the goal of a nationwide program across American College of Cardiology Chapters to amplify cardiovascular prevention for underserved and underrepresented young adults.

## Funding support and author disclosures

A Chapter-Section Grant was received from the 10.13039/100005485American College of Cardiology Chapter of Maryland. Dr Spaulding serves as a consultant to Corrie Health. Drs Ambinder and Goyal have equity in CardioNerds. CardioNerds has received funding from Zoll Lifevest and Pfzer. Dr Martin has received material support from Apple and iHealth; has received funding from the Maryland Innovation Initiative, Wallace H. Coulter Translational Research Partnership, Louis B. Thalheimer Fund, the Johns Hopkins Individualized Health Initiative, the 10.13039/100000968American Heart Association (20SFRN35380046, 20SFRN35490003, COVID19-811000, #878924, and #882415), the Patient-Centered Outcomes Research Institute (ME-2019C1-15 328, IHS-2021C3-24147), the 10.13039/100000002National Institutes of Health (P01 HL108800 and R01AG071032), the 10.13039/100015926David and June Trone Family Foundation, the Pollin Digital Innovation Fund, the PJ Schafer Cardiovascular Research Fund, Sandra and Larry Small, CASCADE FH, Google, and Amgen; and has received personal fees for serving on scientific advisory boards for Amgen, AstraZeneca, Kaneka, NewAmsterdam, Novartis, Novo Nordisk, Sanofi, and 89bio. Dr Marvel has received material support from Apple and iHealth; has received funding from the Maryland Innovation Initiative, Wallace H. Coulter Translational Research Partnership, Louis B. Thalheimer Fund, PJ Schafer Cardiovascular Research Fund, and the 10.13039/100000968American Heart Association Empowered to Serve Business Accelerator. Under a license agreement between Corrie Health and the Johns Hopkins University, the University owns equity in Corrie Health and the University and Drs Martin and Marvel are entitled to royalty distributions related to the Corrie technology. Additionally, Drs Martin and Marvel are founders of and hold equity in Corrie Health. This arrangement has been reviewed and approved by the Johns Hopkins University in accordance with its conflict of interest policies. All other authors have reported that they have no relationships relevant to the contents of this paper to disclose.
